# Kidney disease in nail–patella syndrome

**DOI:** 10.1007/s00467-008-0836-8

**Published:** 2008-06-06

**Authors:** Kevin V. Lemley

**Affiliations:** 1 Division of Nephrology, MS#40, Childrens Hospital Los Angeles, 4650 Sunset Blvd, Los Angeles, CA 90027 USA; 2 Department of Pediatrics, University of Southern California, Keck School of Medicine, Los Angeles, CA USA

**Keywords:** Albuminuria, Creatinine clearance, LMX1B, Homeodomain, Podocyte

## Abstract

Nail–patella syndrome (NPS) is a pleiotropic autosomal-dominant disorder due to mutations in the gene LMX1B. It has traditionally been characterized by a tetrad of dermatologic and musculoskeletal abnormalities. However, one of the most serious manifestations of NPS is kidney disease, which may be present in up to 40% of affected individuals. Although LMX1B is a developmental LIM-homeodomain transcription factor, it is expressed in post-natal life in the glomerular podocyte, suggesting a regulatory role in that cell. Kidney disease in NPS seems to occur more often in some families with NPS, but it does not segregate with any particular mutation type or location. Two patterns of NPS nephropathy may be distinguished. Most affected individuals manifest only an accelerated age-related loss of filtration function in comparison with unaffected individuals. Development of symptomatic kidney failure is rare in this group, and proteinuria (present in approximately one-third) does not appear to be progressive. A small minority (5–10%) of individuals with NPS develop nephrotic-range proteinuria as early as childhood or young adulthood and progress to end-stage kidney failure over variable periods of time. It is proposed that this latter group reflects the effects of more global podocyte dysfunction, possibly due to the combination of a mutation in LMX1B along with an otherwise innocuous polymorphism or mutation involving any of several genes expressed in podocytes (e.g. *NPHS2*, *CD2AP*), the transription of which is regulated by LMX1B.

## Introduction

Nail–patella syndrome (NPS, OMIM 161200) is a pleiotropic autosomal-dominant disorder with complete penetrance but significant variation in its clinical expression [1]. First described as a hereditary disease by Little in 1897 [2], other names for NPS include *h*ereditary *o*nycho-*o*steo*d*ysplasia (HOOD syndrome), Turner–Kieser (or Österreicher–Turner) syndrome, and Fong disease. The general location of the responsible gene was deduced relatively early, due to linkage to the ABO blood group locus [3] and later to the adenylate kinase gene [4] (long arm of chromosome 9).

The incidence of NPS has been reported to be approximately 1:50,000, based on estimates by Renwick many years ago. If the incidence were this high, it would suggest that there should be about 6,000 affected individuals in the USA. The number of detected cases appears to be much lower. Although kindreds of affected individuals may escape diagnosis for generations [5], leading to the possibility of very low rates of case ascertainment, it is also possible that the true incidence of NPS is lower than the historical value of 1:50,000. The classic tetrad of manifestations of NPS reflect dysplasia of structures derived from the dorsal mesenchyme. These include absent or hypoplastic finger- and toe nails; absent or hypoplastic patellae; elbow dysplasia, often involving posterior subluxation of the radial head; and iliac horns. Although iliac horns are pathognomonic of NPS, they occur in only approximately 70% of individuals with NPS [1]. Triangular lunules (or lunulae, the usually crescent-shaped areas at the bases of the finger- and toenails) are very common in NPS. Other common findings in NPS include anterior webbing of the antecubital fossa (pterygium), talipes equinovarus, a lack of creases over the distal interphalangeal joints, cloverleaf pigmentation of the iris (Lester’s sign), primary open-angle glaucoma and ocular hypertension, joint contractures, lumbar lordosis, underdevelopment of proximal limb musculature, attention-deficit hyperactivity disorder, sensory neuropathy, sensorineural hearing loss, irritable bowel syndrome and chronic constipation [1, 6]. Although not part of the defining tetrad of clinical findings, kidney disease occurs in greater frequency in individuals with NPS than in the general population, an association first explicitly noted by Hawkins and Smith [7] in 1950, although clearly described by Kieser in 1939 [8]. NPS was recently reviewed in *Pediatric Nephrology* [9].

### Molecular genetics of NPS

Despite the early discovery of linkage of NPS to the ABO blood group locus, the specific gene responsible for NPS was only identified in the late 1990s. *Lmx1b* (the mouse ortholog to the human gene, *LMX1B*) is one of a family of more than nine LIM-homeodomain genes regulating gene transcription via its interactions with gene promoter and enhancer sequences, in conjunction with other transcription factors. *Lmx1b* and its ortholog in the chicken (*Lmx-1*) were previously known to be involved in dorsoventral limb patterning. A knockout of *Lmx1b* in the mouse resulted in a homozygous phenotype that was highly suggestive of NPS, albeit manifesting neonatal lethality [10]. The mouse gene *Lmx1b* maps to a region syntenic to the known location of the NPS gene in humans (chromosome 9q34). Based on this finding, this region was sequenced directly in three patients with NPS, and three different de novo mutations were found in *LMX1B* [11]. NPS was the first human genetic condition associated with a mutation in a LIM-homeodomain gene. Another small study described four novel mutations in four families [12]. In a study of 41 families with NPS, 25 mutations in *LMX1B* were found by DNA sequencing and gel analysis in 37 of the families [13]. These included frameshift mutations, single-base deletions, larger deletions/insertions and single-base substitutions (leading to nonsense, splice and missense mutations). Balanced translocations have also been reported [14]. In families without identified mutations, the relevant DNA changes are thought to lie in promoter or intronic regions. Currently, more than 130 distinct mutations in *LMX1B* have been described. Most of these are in the LIM domains (∼80%), with a smaller number in the homeodomain (∼20%) [9] and a small number in the C-terminal region of the gene [14]. No mutation is found in 10–15% of patients investigated. Approximately 12.5% of cases are sporadic (no demonstrable family history) [1].

The gene is expressed in a number of tissues implicated in the clinical phenotype in the fetus. In the kidney, it is expressed in podocytes from the S-shaped body stage on into post-natal life [10]. Of adult organs, in fact, as shown by the Northern blot technique, only the kidney expresses *LMX1B* [11]. In the nephron, only the glomerulus expresses the gene [15].

The protein product LMX1B consists of two cysteine-rich Zn-binding LIM domains in the N-terminal portion, a 60-amino acid homeodomain (HD) and a C-terminal activation sequence rich in glutamine and serine residues. The LIM domains underlie protein–protein interactions with other transcription factors and modifiers [such as the LIM domain binding protein, Ldb1 (also known as the cofactor of LIM domains or CLIM2) [16] or basic helix–loop–helix (bHLH) proteins]; the HD is necessary for binding of specific promoter and/or enhancer sequences of DNA (e.g. via the FAR/FLAT sequence element). Missense mutations in the HD decrease binding to target (FLAT) sequences [13]. There is significant sequence homology to LMX1B in other species: 99% amino acid sequence identity is found among mammals, and even 87% identity between mammals and teleosts [6]. For this reason HD segments of human and non-human mammalian orthologs of LMX1B bind similarly to target DNA sequences in various test systems, so function of the human gene can, in principle, be deduced from the behavior of non-human orthologs.

The gene *LMX1B* contains eight exons. The transcription start site is downstream of a cluster of CG-rich sequences (CpG islands) and is not associated with a consensus TATA box. This is typical of genes that are transcribed at a low rate, often under control of the transcription factor Sp1 [14]. With respect to its promoter structure, basal transcription of *LMX1B* requires the nucleotide bases from 112 bases upstream of the transcription start site to 807 bases downstream from it (i.e. nt −112/+807). There are, however, two open reading frames (ORFs) in the presumptive 5′-untranslated region (5′-UTR) of the gene, one with the potential to code for an additional 23 highly conserved amino acids in-frame with the presumed transcription start site. Owing to alternative splicing of the last 21 nucleotides in exon 7, the predicted protein sequence is 372 or 379 amino acids (395 or 402 amino acids including the 23-amino acid 5′-UTR sequence) [14].

A number of target genes for LMX1B have been described. The LMX1B product binds to the putative enhancer sequence of *COL4A4* (in the common regulatory region responsible for coordinate expression of *COL4A4* and *COL4A3* [17], two of the three genes required for production of mature type IV collagen in the glomerular basement membrane, GBM). The mouse knockout Lmx1b−/− has strongly decreased expression of α(3) and α(4) chains of type IV collagen, while the heterozygous Lmx1b+/− has moderately decreased expression of the relevant mRNA species [18]. On the other hand, expression of all three chains (α3, α4, α5) of type IV collagen is shown to be normal by immunohistochemical analysis in the glomeruli from biopsies of humans with NPS [19]. Expression of the podocyte-related genes *CD2AP* and *NPHS2* is also reduced in the *Lmx1b*−/− mouse [17, 20], although, again, not in affected humans [19]. Each of these genes has FLAT elements flanking the first exons. However, although Lmx1b binds a FLAT element in the *NPHS2* promoter, there have been conflicting reports regarding its ability to activate a reporter gene on that promoter [17, 20], possibly due to the lack of an essential transcription cofactor in some, but not all, of the expression systems used.

LMX1B is thought to interact with other gene regulatory proteins: negatively with Ldb1 and positively with the bHLH protein, E47 [21]. The bHLH transcription factor *Pod1* is expressed in parallel fashion with *Lmx1b* in the kidney, but Pod1 and Lmx1b have not been shown to interact as transcriptional cofactors [22]. With its two LIM domains, LMX1B is likely to have multiple regulatory protein ‘partners’. The renal development transcription factor, PAX2, for example, has been shown by Marini and colleagues [23] to interact with LMX1B only in the presence of the latter’s HD (inasmuch as deletion of the HD abrogated the interaction, whereas deletion of the LIM domains did not). This suggests that a structural change in LMX1B upon DNA binding may facilitate its interaction with other proteins.

In any autosomal dominant condition, different possibilities exist for the mechanism by which mutation in a single allele alters aggregate gene function. In the case of *LMX1B*, haplo-insufficiency or a dominant negative effect have been the mechanisms most often discussed. There is also the theoretical possibility of a gain-of-function mutation (e.g. as in hypoxia-inducible factor-α, HIFα). In the dominant negative mechanism, an abnormal gene product interferes with the normal actions of the wildtype (WT) product in the same cell, either by forming a non-functional dimer (if, for example, the functional gene product is a homodimer) or by strongly and competitively binding to, but not activating, the target DNA sequence. This mechanism often leads to a more severe phenotype than a simple null mutation. In various in vitro systems, co-expression of both mutant and WT Lmx1b did not interfere with activation of a reporter construct by the WT product [21, 24], suggesting haplo-insufficiency as the fundamental mechanism. It is, as yet, unclear why NPS shows autosomal dominant genetics in humans and an autosomal recessive pattern in the mouse. A recent study of a podocyte-specific *Lmx1b* knockout (using a Cre-Lox system based on the *NPHS2* promoter) showed later development of proteinuria and greater expression of type IV collagen chains and podocin, although the animals still died of kidney failure within 14 days [15]. Thus, even this model still represents a more severe kidney phenotype than in the human disease.

The presence of significant phenotypic variation in a presumably monogenic disorder makes NPS an attractive subject for both clinical and basic investigations. Over 50 years ago, Renwick suggested that the NPS allele of the *unaffected* parent was responsible for variations in disease severity (as manifested in an orthopedic ‘score’ of nail or patellar signs). Recently, Dunston and colleagues [25] examined this modifier allele effect in 103 individuals, using a similar quantitative nail scoring system and single nucleotide polymorphism (SNP) haplotype profiling to establish allelic inheritance. Contrary to the findings and interpretation of Renwick [26], those authors found evidence that the NPS allele of the affected parent (but not the specific mutation) influenced the severity of nail dysplasia, suggesting the influence of a *cis*-acting element near the mutant gene. As yet, there has been no study associating SNP haplotypes to either the presence of nephropathy or quantitative proteinuria, analogous to Dunston’s work on nail dysplasia.

### Clinical manifestations

Because of the rarity of the disorder, most publications concerning kidney involvement in NPS have either been case reports or small series. Involvement of the kidney as a feature of NPS was first reported in 1950 [7]. Hematuria and proteinuria have often been noted, as have numerous anecdotal reports of progression to kidney failure, even in childhood [27]. In fact, the reported cases of kidney failure do not seem to conform to the typical progressive pattern of other genetic diseases, such as Alport syndrome. Other renal abnormalities (urologic malformations, nephrolithiasis) have been reported anecdotally [27–29] but have not been investigated systematically.

Only a limited number of larger series (involving 36 to 236 individuals) addressing kidney involvement in NPS have been reported [1, 27, 28, 30–32], and there have been no longitudinal studies on kidney involvement in NPS. The larger studies represent patients from a single center [28], from a systematic cross-sectional multi-site survey [1] or collected from multiple centers and published reports [27, 30–32]. Thus, ascertainment of subjects has always been potentially biased towards more severe referral cases; the closest to an unbiased ascertainment is probably in the reports of Sweeney and colleagues [1] and Bongers and colleagues [31]. In the five largest series [1, 27, 30–32], screening for kidney involvement involved the questioning of subjects and simple urinalysis, spot urine protein(albumin)/creatinine ratios and/or estimated glomerular filtration rate (GFR), or it could not be deduced from the description of the methods in the published reports. Among the larger series, hematuria was only specifically reported in three studies [1, 27, 31]. In these the incidence was approximately 10–20%. The presence of elbow pterygia has been reported to be associated with the presence of nephropathy in NPS [33], but this was not substantiated in a large study [1].

In the study of 36 affected individuals by Bennett and colleagues [28], in addition to a dipstick urinalysis, 4 h creatinine clearance was done as a part of the screening. Follow-up determinations of urinary protein excretion were performed only in those individuals having a positive result for the urine dipstick test for protein. Nineteen of the 36 NPS subjects underwent more extensive investigations, including that of 24 h creatinine clearance and quantitative proteinuria determinations. Of these, only one subject (a 21-year-old woman with proliferative glomerulonephritis) had a creatinine clearance less than 75 ml/min per 1.73 m^2^ body surface area. Two subjects (both children) had nephrotic syndrome, and three adults had moderate proteinuria (130–350 mg/day). The incidence of hypertension was not mentioned in that study.

In the 1988 study of Looij and colleagues [30] of approximately 240 affected individuals, the risk of nephropathy in 118–131 subjects having a parental history of NPS nephropathy (47–59%) was about the same as that of the group as whole (48%). Similarly, the risk of kidney failure was the same in individuals with (15%) and without (14%) a parental history of NPS and kidney failure. Meyrier and colleagues [27] reported that, of 123 patients with NPS with adequate information, 62% had some sign of kidney involvement (proteinuria, hematuria, hypertension or kidney failure); of these, approximately 15% had developed kidney failure. They also reported on a pair of identical twins with NPS, one of whom progressed rapidly to kidney failure, while the other remained “only proteinuric”. Sweeney and colleagues [1] studied 123 British NPS patients (ages 4 months to 80 years) from 43 families. The patients were ascertained via clinical genetics departments, patient contact groups and nephrologists. The authors found an overall incidence of kidney involvement (as determined by urine dipstick analysis and clinical history) of 37.5%. With the exclusion of those subjects with clinical findings only during pregnancy, the rate was 25%. Only 3% had developed kidney failure, whereas the incidence of pre-eclampsia in women with NPS was 29%, approximately ten-times the incidence in the general population [1]. Other, isolated, cases of pre-eclampsia in NPS have been reported [34]. Knoers and colleagues [32] investigated NPS genotypes in eight affected Dutch families (66 individuals; sporadic in three families). The subjects had some overlap with those reported earlier by Looij and colleagues in 1988. The authors expressed doubt that the high incidence of kidney disease in the largest of the families (present in 13 of 30 individuals, reported previously in [30]) could be related to the familial LMX1B genotype, inasmuch as the family contained affected individuals with no evidence of kidney disease, individuals with mild proteinuria alone and individuals whose condition had progressed to kidney failure. Of interest, that family’s specific genotype (the splice-site mutation 672 + 1 G→A) was not associated with occurrence of nephropathy in an unrelated kindred [13]. Finally, in a recent study of 106 individuals from 32 families [31], some of whom had been reported on in previously published studies from the same center [30, 32], urinary studies were performed on 81 subjects ranging from 6 months to 82 years of age. Microalbuminuria was present in 27.5% of morning urine specimens, while macroalbuminuria (defined as > 300 μg albumin/10 mM creatinine) occurred in 3.6%. Estimated GFR was < 60 ml/min per 1.73 m^2^ in 5.3% and between 60 ml/min per 1.73 m^2^ and 90 ml/min per 1.73 m^2^ in 34.2%. The authors estimated GFR using the formula of Cockroft and Gault. It must be kept in mind that a low relative muscle mass, typical in individuals with NPS [1], may lead to lower creatinine production rates and spuriously elevated renal function as estimated by the Cockcroft–Gault and other equations. Unlike the previous interpretation of their data, in this report the authors proposed that nephropathy was more common in individuals with mutations in the HD region. Acknowledging the overriding influence of the single large family mentioned above, they stated that a significant effect of genotype on nephropathy risk was still present when this family was removed (53% of the variation in proteinuria was attributable to family and 30% to LMX1B genotype). The incidence of hypertension was not mentioned in their report. They did report that proteinuric subjects were older than non-proteinuric subjects (48  years vs 36 years, *P* = 0.03) and that the GFR in proteinuric subjects was less than in non-proteinuric subjects (78 ml/min per 1.73 m^2^ vs 100 ml/min per 1.73 m^2^).

It seems likely that many of the earlier studies of kidney involvement in NPS may not have identified all individuals with low-grade proteinuria. Results of standard dipstick tests are only reliably positive for proteinuria in urine specimens having albumin/creatinine ratios over approximately 300 μg/mg, whereas urinary albumin excretion in healthy individuals corresponds to a ratio less than 30 μg/mg. Thus, dipstick screening will not detect some individuals with low-grade albuminuria. The importance of the detection of proteinuria in the occult, microalbuminuric stage (spot urinary albumin/creatinine ratios from 30 μg/mg to 299 μg/mg) has been emphasized principally in patients with type 1 diabetes, as diabetic individuals with persistent microalbuminuria have a substantially increased risk of developing overt nephropathy over time [35, 36]. The same has not been demonstrated in NPS.

The lack of longitudinal studies of kidney involvement in NPS makes it difficult, if not impossible, to determine precisely which clinical features characterize progression to end-stage kidney failure. For example, it is, as yet, unknown if such patients always manifest nephrotic-range proteinuria prior to progression to kidney failure.

### Pathology of NPS in the kidney

In the animal model of NPS (the Lmx1b−/− mouse) the podocytes and glomeruli seem to have arrested development, with capillary ingress but not arborization. Podocytes lack normal foot processes and slit diaphragms (adherens junctions may be present instead, between podocytes) [17, 20]. The glomerular tuft is small, the GBM is split, the glomerular endothelial cells have decreased fenestrae, and fibrin-like material may be deposited in Bowman’s space [20]. In addition to these findings, lifting of glomerular capillary endothelial cells off the GBM has been described in mice with podocyte-specific homozygous deletion of *Lmx1b* [15], which also present with later-onset kidney injury and death. Staining for some podocyte-related proteins [CD2-associated protein (CD2AP), podocin], but not others (nephrin, synaptopodin), is significantly decreased [20, 37].

**Fig. 1:**
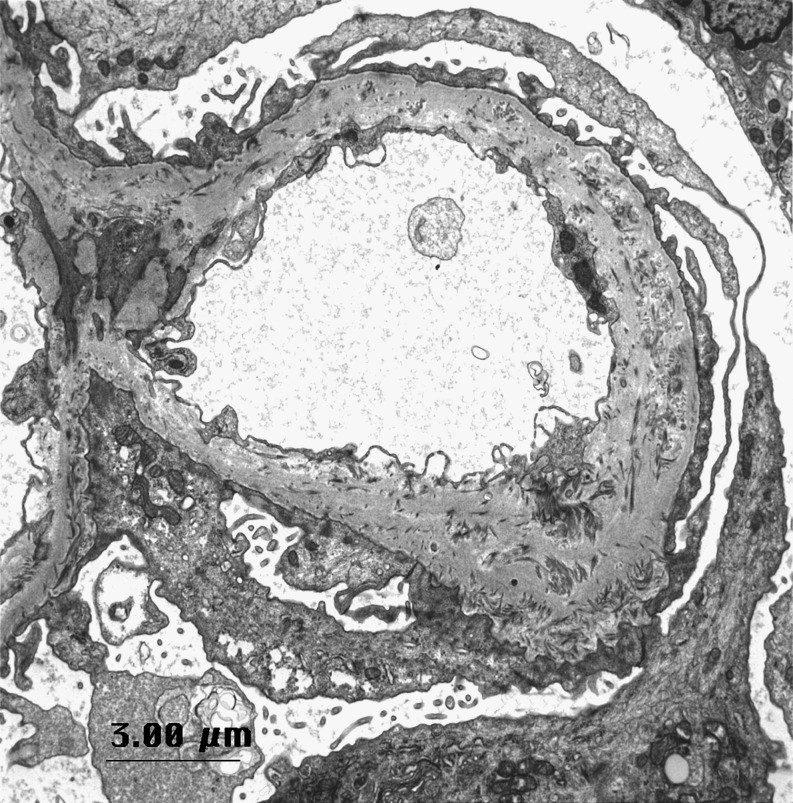
Electron micrograph from patient with NPS and nephrotic-range proteinuria. Notice the extensive effacement of podocyte foot processes and the irregular thickening of the glomerular basement membrane with scattered deposits of collagen fibrils. Stained with lead citrate and uranyl acetate

The first description of ultrastructural abnormalities in the NPS kidney was published in 1970 [38]. On light microscopy, the glomeruli of individuals with NPS are not, in general, remarkable, with the exception of variable amounts of glomerular sclerosis. On electron microscopy, patients with NPS show characteristic irregular thickening of the glomerular basement membrane, including deposits of bundles of striated type III collagen fibers in the lamina densa [19] and patchy lucent (“moth-eaten”) areas (Fig. 1). Fibrillar collagen bundles are also occasionally seen in the mesangial matrix. Staining with phosphotungstic acid may improve visibility of these collagen bundles over routine uranyl acetate/lead citrate staining. Foot processes are normal or focally effaced [19]. There are few reports commenting on the presence or abnormality of slit diaphragms in NPS patients. In the study by Heidet and colleagues [19], slit diaphragms are simply reported as being “preserved”. Immunofluorescence microscopy gives negative findings or shows non-specific staining for IgM, C3 and/or C1q, often in sclerotic portions of the tuft. Fibrin deposition along the GBM may be a rare finding in humans [28]. Unlike the homozygous mouse model, in patients with NPS the glomeruli stain normally for the α3 and α4 chains of type IV collagen, podocin, synaptopodin, glomerular epithelial protein 1 (GLEPP1), CD2AP, α3 integrin and nephrin.

NPS in the kidney must be distinguished from collagen type III glomerulopathy [39]. The latter autosomal recessive hereditary nephropathy has distinct systemic manifestations and demonstrates widespread deposition of type III collagen within the mesangial matrix and along the subendothelial aspect of a normal lamina densa of the GBM. Numerous reports exist of NPS patients having biopsy diagnoses of other renal diseases (Goodpasture syndrome, IgA nephropathy, membranous nephropathy, hemolytic uremic syndrome), although the significance of these anecdotal associations is uncertain. Typical ultrastructural findings of NPS may be found in individuals with no clinical evidence of kidney involvement, and the severity of specific pathologic findings seems largely unrelated to the clinical severity or prognosis [28]. On the other hand, a patient with long-standing hematuria and proteinuria and typical findings of NPS on renal biopsy by report did not have any nail or skeletal findings of NPS [40]. No mutation analysis was provided in this report. There have been no reported series of repeat biopsies of patients with NPS to answer such questions as to whether the typical GBM abnormalities are progressive.

### Diagnosis of NPS

A diagnosis of NPS is suggested by the usual tetrad of musculoskeletal features: absent/dysplastic nails, absent/hypoplastic patellae, elbow dysplasia or radial subluxation, iliac horns. Although diagnosis can be made at birth, it is often missed, presumably due to the rarity of the condition and its sometimes subtle clinical forms. Owing to the phenotypic variability of NPS, it is advisable that a clinical geneticist be consulted to confirm a suspected diagnosis. Pre-natal (first trimester) diagnosis is possible using chorionic villus sampling, either with marker haplotyping or kindred-specific mutation analysis [41]. Commercial gene sequence analysis is also available from multiple laboratories in the USA and abroad (see www.genetests.org for contact information).

Biopsy to investigate the possibility of the presence of another kidney disease in patients with NPS could be justified if signs and symptoms of another form of nephropathy are also present (lupus, diabetic nephropathy). As there is no clear relationship between the renal pathology of NPS and its clinical behavior, kidney biopsy in cases of NPS with classical features is to be discouraged.

### Care for individuals with NPS

Owing to the rarity of NPS, many affected individuals find it difficult to obtain comprehensive care from their primary care provider, who is likely to be completely unfamiliar with the multiple clinical manifestations of NPS when the patient first walks into the office. It is often a subspecialist (orthopedist, nephrologist, geneticist) who eventually coordinates care. The treating physician should carefully balance investigating every possible clinical component of the disease state and blindly ascribing any (possibly unrelated) clinical finding to NPS.

It has been suggested that screening for nephropathy be done annually, starting at birth. In the absence of other signs of nephrotic syndrome, as well as any evidence that early intervention affects clinical outcome, it seems likely that deferring annual screening until a child can reliably give a specimen by spontaneous voiding (“clean catch”) may be an acceptable strategy. Although spot urine protein/creatinine or albumin/creatinine ratios are more sensitive than routine urinary dipstick analysis, there is, as yet, no evidence that intervention in the microalbuminuric phase will alter outcome. Other routine screening is ophthalmology examination for detection of glaucoma.

### Treatment of the nephropathy of NPS

Progressive kidney disease affects a small minority of patients with NPS: estimates range from 3% [1] to 15% [27, 30]. Significant questions remain concerning the prognosis of individuals detected with abnormal albuminuria and a normal GFR. Nephrotic-range proteinuria has often been associated with loss of kidney function over variable periods of time. Patients with proteinuria and long-term stability of kidney function have also been mentioned in the literature (although usually without much clinical data). As it is a genetic disease, not surprisingly, there are no specific treatments for kidney involvement in NPS. A strongly renoprotective effect of angiotensin-converting enzyme (ACE) inhibition on disease progression has been demonstrated in a variety of non-diabetic, proteinuric, glomerular diseases [42], raising the possibility that treatment with ACE inhibitors might provide equivalent protection to proteinuric patients with NPS (vide infra). In the author’s personal experience, treatment with low-dose ACE inhibitors in an 8-year-old child with NPS reduced the urine protein/creatinine ratio from 2.2–2.8 mg/mg to 0.4–0.8 mg/mg. It seems likely that the development of national or multinational disease registries for NPS will be necessary in order to follow effectively the relatively small number of NPS patients with known kidney involvement and allow the acquisition of the longitudinal data needed to evaluate both potential prognostic markers (e.g. proteinuria) and possible therapeutic interventions such as ACE inhibition.

A novel, and unexpected, therapy has been described in recent years for another genetic disease characterized by abnormalities of the GBM, Alport syndrome. Cyclosporin has been described in some, but not all, reports as decreasing proteinuria and stabilizing kidney function [43, 44]. Although no studies have addressed its use in NPS with high-grade proteinuria, a trial of cyclosporin may be a reasonable option for individuals whose condition is resistant to the anti-proteinuric action of ACE inhibitors or in whom unacceptable side effects (hypotension, angioedema) develop.

There are numerous anecdotes of patients with NPS who have undergone successful transplantation after developing terminal kidney failure. A normal distribution of type IV collagen and various podocyte proteins in the human disorder may explain why there is apparently no development of (antibody-mediated) neo-antigen disease in transplant recipients. Generally, patients with NPS and kidney failure should be considered excellent candidates for kidney transplantation. NPS should, of course, be excluded from potential related kidney donors.

### The Pittsburgh study

As stated above, there have been no published longitudinal studies of kidney function in patients with NPS. In 2002, my colleagues (Adele Towers, Ketki Desai and Iain McIntosh) and I undertook a study of various aspects of the NPS phenotype, including kidney function. By examining the effects of age on kidney function in a large cross-sectional study, we simulated longitudinal changes in NPS, using each individual as a surrogate for a temporal cross-section of a “global (corporate) individual”. The participants at the 2002 national conference of the patient support organization Nail–Patella Syndrome Worldwide (NPSW, www.nailpatella.org) were invited to participate in a study approved by the Institutional Review Board of the University of Pittsburgh School of Medicine. Twenty-five children (ages 2–17 years) and 32 adults (ages 21–69 years) with a diagnosis of NPS confirmed by a clinical geneticist were enrolled, representing 29 kindreds. Participants underwent measurement of blood pressure, serum creatinine and urine albumin/creatinine ratio (not all measurements were obtained in all individuals). A study of bone health in this group has been reported [45]. GFR in the children was estimated from the Schwartz formula [46], and, in the adults, creatinine clearance was estimated by the Cockcroft–Gault formula, corrected for body surface area (BSA), as described before [31]. Normal values for adults were derived from data from 138 healthy, age-matched unaffected individuals (21–69 years) previously studied at Stanford University (unpublished observations). *LMX1B* mutation analysis was performed as previously described [13]. Mutations were characterized as null, missense LIM or missense homeodomain. None of the subjects had developed end-stage kidney disease. One child and two adults were taking ACE inhibitors.

Hypertension was rare (none of 19 children, two of 28 adults, had mild stage 1 hypertension). Six of 17 children and eight of 30 adults had proteinuria. There was no relationship between age and albumin/creatinine ratio in the adults (*P* = 0.997). All children had a normal estimated GFR (*n* = 10; 156 ± 24 standard deviation (SD) ml/min per 1.73 m^2^ BSA). In 26 adults, the estimated creatinine clearance, corrected for BSA, was 93 ± 24 ml/min per 1.73 m^2^. The control adults had a higher average creatinine clearance (113 ± 21 ml/min per 1.73 m^2^, *P* < 0.001). The rate of decline of the creatinine clearance with age in the adults with NPS was 1.15 ± 0.33 standard error (SE) ml/min per 1.73 m^2^ per year (*P* = 0.002) compared to 0.50 ± 0.13 ml/min per 1.73 m^2^ per year (*P* = 0.0002) in controls. The control value was quite similar to the rate of decline of measured 24 h creatinine clearance with age (0.50–0.62 ml/min per 1.73 m^2^ per year) previously described in healthy men [47, 48].

**Fig. 2:**
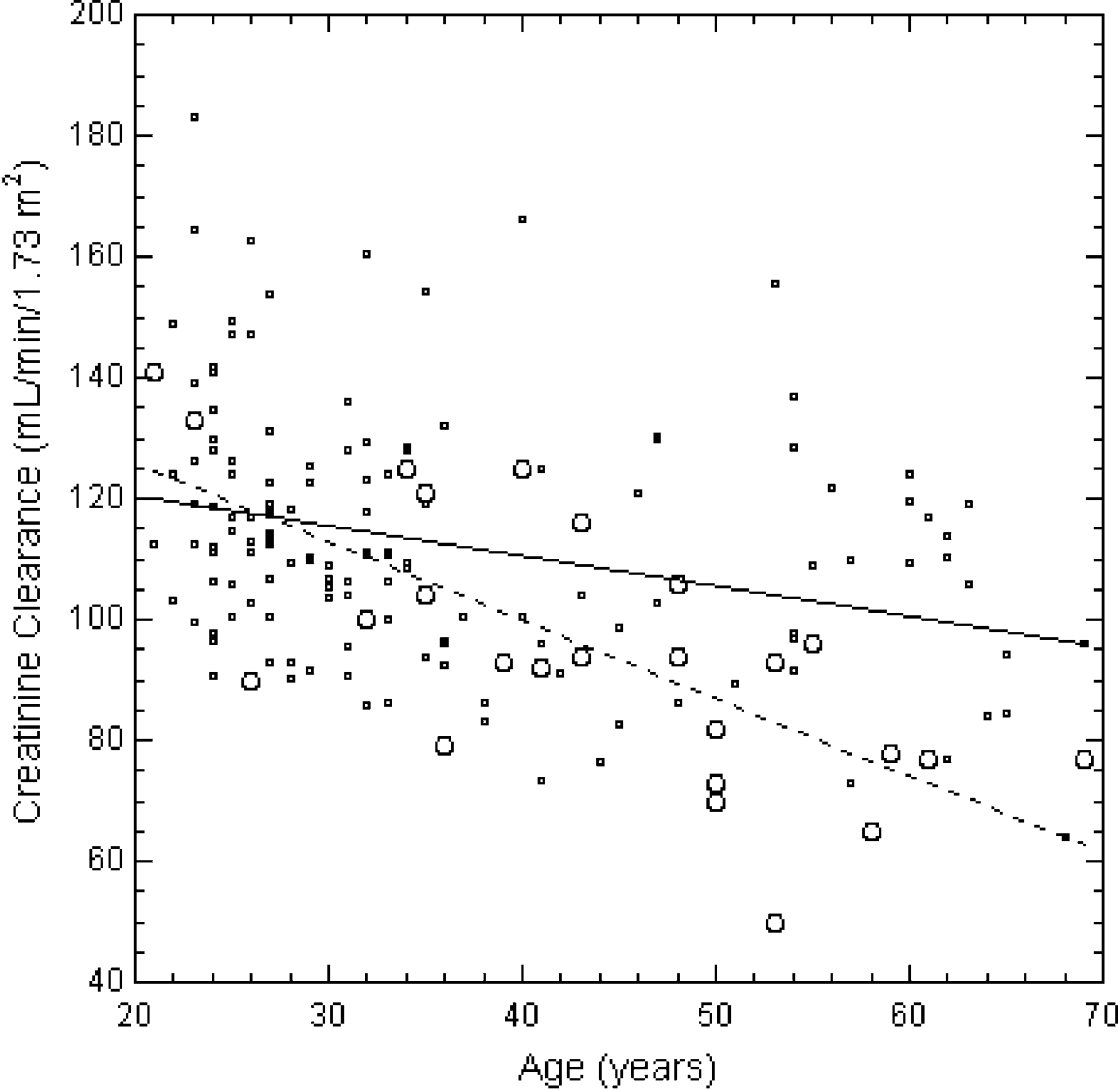
Creatinine clearance age-matched controls (*small circles*, *solid line*, *n* = 138) and 25 adult subjects with NPS (*large circles*, *dashed line*). The slopes of the regressions differ significantly (0.50 ± 0.13 vs 1.29 ± 0.28 SE ml/min per 1.73 m^2^ per year; *P* = 0.018)

To examine the effects of *normal* aging on kidney function in NPS, we looked at the relationship between creatinine clearance and age in affected individuals who did not have overtly decreased kidney function. Although two subjects had decreased age-specific creatinine clearances when compared to the reference unaffected population (a 35-year-old woman with a clearance of 48 ml/min per 1.73 m^2^ and a 53-year-old man with a clearance of 50 ml/min per 1.73 m^2^), we removed from the analysis only that subject whose creatinine clearance fell outside the 95th percentile range of the regression of creatinine clearance on age among the individuals with NPS; that is, we let the regression relationship define the normal rate of change of creatinine clearance with age in NPS. The decline in creatinine clearance in the remaining 25 subjects with NPS, 1.29 ± 0.28 SE ml/min per 1.73 m^2^ per year, was significantly greater than that in the controls (*P* = 0.018) (Fig. 2).

Kidney involvement seemed to be differently distributed among the various NPS mutations. Twenty-five different mutations were included in our sample, representing approximately 18% of those mutations that have been previously described [14]. Of those mutations present in two or more individuals, kidney involvement (defined as decreased creatinine clearance, proteinuria or hypertension) seemed to occur with increased frequency in c.819 + 1G →A (two of two), p.C146Y (two of three) and p.L252P (three of three). In this terminology, c.819 + 1G→A means the substitution of an adenosine for a guanosine at the first nucleotide of the intron that follows the cDNA (c.) position 819; p.C146Y means a change in the 146th amino acid of the protein (p.) from cysteine to tyrosine (Y) [49]. A decreased frequency of kidney involvement seemed to be a feature of mutations p.Q60X (one of nine), p.R221X (none of four), and p.R231X (none of six). Statistical examination of the 11 mutations represented by more than one individual suggested non-random clustering of affected individuals among the different mutations (χ^2^ = 24; *P* = 0.008). The three most common mutations associated with kidney involvement, however, were each of a different type: c.819 + 1G →A is a null mutation; p.C146Y is a missense mutation in the LIM-B domain; p.L252P is a missense mutation in the homeodomain. Thus, as with the study by Knoers and colleagues [32], although there was evidence for familial aggregation of nephropathy, we found no convincing evidence for a specific “nephropathy locus” in LMX1B.

### NPS nephropathy: a possible mechanism

As NPS is a genetic disease, it is clear that its clinical manifestations are a result of mutations in or around the gene *LMX1B*. Since kidney function exists at the level of the organ (or at least its major structural components, the glomerulus and the tubule), the renal manifestations of NPS belong to the realm of physiology and not of molecular biology. In short, we ultimately need to address how we get from gene to GFR. Nephropathy in NPS (both in terms of proteinuria and loss of GFR) seems to be characterized by rather extreme phenotypic variability. I propose that kidney involvement in NPS can be understood in terms of two distinct mechanisms. First, the vast majority (90–95%) of individuals with NPS have a slowly progressive loss of filtration function with age, at approximately twice the rate associated with normal aging in unaffected individuals. This is likely due to progressive thickening and disorganization of the GBM, lowering the hydraulic permeability of the glomerular capillary wall [50]. No pathology studies address whether the thickening of the GBM in NPS is progressive. There is low-grade, non-progressive, proteinuria in a minority of these individuals, suggesting that global podocyte dysfunction is not present. A small proportion (5–10%) of patients manifest greater amounts of proteinuria and tend to have a progressive, albeit variable, course to kidney failure. It seems possible that these individuals have, in addition to the single heterozygous *LMX1B* mutation, another heterozygous mutation or polymorphism in the exons, promoter or enhancer elements of a gene pivotal to podocyte function (e.g. *NPHS1*, *NPHS2*, *CD2AP*, *ACTN4*), the expression of which is regulated by LMX1B. The latter mutation/polymorphism alone would not lead to a renal phenotype, but, in conjunction with haplo-insufficiency in *LMX1B*, it leads to varying degrees of podocyte dysfunction. The variability of the rate of progression may result from the particular combination of podocyte gene changes. This could certainly explain the increased, but not universal, occurrence of more severe kidney involvement within some families (but not with specific *LMX1B* mutation types), as these other mutations/polymorphisms would be expected to segregate independently from *LMX1B* (e.g. *NPHS2* is on chromosome 1). It is intriguing in this regard to consider the report [51] that a neonate in Finland with NPS had transient nephrotic syndrome at birth. It seems possible that this was the effect of heterozygous mutations in *LMX1B* and *NPHS1,* although the transient nature of the nephrosis is difficult to explain under this model.

If the age-related loss of filtration function in most adults with NPS is not due to global podocyte dysfunction (as suggested by the lack development of progressive proteinuria) but rather to thickening of the GBM, it is open to question as to how effective ACE inhibition would be in the stabilization of glomerular filtration function in these individuals with NPS. On the other hand, anecdotal experience suggests that these individuals are not at high risk for developing end-stage kidney failure over the course of a normal lifetime. Thus, it is to the relatively small number of NPS patients with either nephrotic-range proteinuria or evidence of significant loss of GFR that we should direct our efforts to find an effective treatment.

## Questions

(Answers appear after the reference list)

1. Which of the following is not one of the classic tetrad of diagnostic features of NPS:
  A. dysplastic finger or toe nails
  B. elbow dysplasia
  C. low-set ears
  D. hypoplastic or absent patellae
2. The gene responsible for NPS—LMX1B—is
  A. expressed only after birth
  B. on the X chromosome
  C. a LIM-homeodomain transcription factor
  D. quite different in sequence from its non-human mammalian orthologs
3. Kidney failure in NPS is
  A. associated with sensorineural hearing loss
  B. rare under the age of 40 years
  C. relatively uncommon (5–10% of individuals)
  D. associated with more severe changes of the GBM on biopsy
4. Some sign of kidney involvement (hematuria, proteinuria, increased creatinine) in NPS is found in
  A. < 10% of individuals
  B. 10–20% of individuals
  C. 30–40% of individuals
  D. 100% of individuals
5. The pathology of NPS in the kidney is characterized by
  A. diffuse IgG deposits in the mesangium
  B. fibrillar collagen in the lamina densa of the GBM
  C. its close prognostic connection to functional outcome
  D. being non-specific for this disorder

## Abbreviations

bHLH: Basic helix-loop-helix
HD: Homeodomain
HIFα: Hypoxia-inducible factor-α
ORF: Open reading frame
5′-UTR: 5′-Untranslated region
WT: Wildtype
